# Corneal ring infiltrate- far more than Acanthamoeba keratitis: review of pathophysiology, morphology, differential diagnosis and management

**DOI:** 10.1186/s12348-023-00379-6

**Published:** 2023-12-19

**Authors:** J. Przybek-Skrzypecka, J. Skrzypecki, L. Suh, J. P. Szaflik

**Affiliations:** 1 Department of Ophthalmology, Medical University of Warsaw, Marszałkowska 24/26, 00-576 Warsaw, Poland; 2 SPKSO Ophthalmic University Hospital, Warsaw, Poland; 3 Department of Experimental Physiology and Pathophysiology, Medical University of Warsaw, Warsaw, Poland; 4 Cornea & Refractive Surgery, Edward S. Harkness Eye Institute, Columbia University Irving Medical Center, New York, USA

**Keywords:** Corneal ring infiltrate, Sterile corneal infiltrate, Wessely ring, Keratitis, Acanthamoeba

## Abstract

**Objective:**

Ring infiltrates usually accompany numerous infectious and sterile ocular disorders. Nevertheless, systemic conditions, drugs toxicity and contact lens wear may present with corneal ring infiltrate in substantial part. Considering its detrimental effect on vision, detailed knowledge on etiology, pathophysiology, differential diagnosis, and management should be considered essential for every ophthalmologist.

**Methods:**

The PUBMED database was searched for “corneal ring infiltrate” and “ring infiltrate” phrases, “sterile corneal infiltrate” and “corneal infiltrate”. We analyzed articles written in English on risk factors, pathophysiology, clinical manifestation, morphological features, ancillary tests (anterior-segment optical coherence tomography, corneal scraping, in vivo confocal microscopy), differential diagnosis and management of corneal ring infiltrate.

**Results:**

Available literature depicts multifactorial origin of corneal ring infiltrate. Dual immunological pathophysiology, involving both antibodies-dependent and -independent complement activation, is underlined. Furthermore, we found that the worldwide most prevalent among non-infectious and infectious ring infiltrates are ring infiltrates related to contact-lens wear and bacterial keratitis respectively. Despite low incidence of Acanthamoeba keratitis, it manifests with corneal ring infiltrate with the highest proportion of the affected patients (one third). However, similar ring infiltrate might appear as a first sign of general diseases manifestation and require targeted treatment. Every corneal ring infiltrate with compromised epithelium should be scraped and treat as an infectious infiltrate until not proven otherwise. Of note, microbiological ulcer might also lead to immunological ring and therefore require anti-inflammatory treatment.

**Conclusion:**

Corneal ring infiltrate might be triggered not only by ocular infectious and non-infectious factors, but also by systemic conditions. Clinical assessment is crucial for empirical diagnosis. Furthermore, treatment is targeted towards the underlying condition but should begin with anti-infectious regimen until not proven otherwise.

## Background

“Corneal infiltrates” and “corneal ulcers” are one of the most common emergency issues, both in Acute and Emergency Eye Division (A&E) and the corneal specialists’ office [1]. Although it is of the highest importance to properly assess the origin and implement the right treatment, direct diagnosis is far from straightforward. Dalmon et al. proved that even experienced corneal specialists can properly identify the etiology of corneal ulcers in 66% of cases based solely on anterior segment slit-lamp photographs [2]. Similar, or even higher, uncertainty about the etiology arises when the special type of corneal infiltrate termed “ring infiltrate” is present.

Corneal ring infiltrate (CRI), by definition, is a ring-shaped stromal infiltrate of 360 degrees, circumferential to the limbus, typically leaving a clear zone from it. Ring infiltrates may originate from both infectious and sterile processes [3]. The existing body of evidence associates CRI mainly with Acanthamoeba keratitis and fungal keratitis in their late stages, but the list of potential origins substantially exceeds the aforementioned causes [4].

Despite the well-established definition and morphology, several questions about appropriate differential diagnosis and management of CRI arise. If corneal scraping is the gold standard ancillary test for each CRI differentiation? Which laboratory tests should be performed? Can anterior segment optical coherence tomography (OCT), in vivo confocal microscopy (IVCM), general physical examination, and blood tests also be helpful in diagnosis? Do these infiltrates appear when patients are symptomatic from a systemic process or are they the first symptoms of the disease? How does the prevalence of underlying disease change the perspective of ancillary testing performed when CRI is present. Does the depth of the ring suggest etiology and affect final visual outcome?

This review aims to provide a comprehensive analysis of potential origins of corneal ring infiltrate, characteristic of their morphological features, ancillary testing useful in differential diagnosis, the importance of a medical history, and consideration of general diseases and medications. Moreover, we attempt to differentiate between corneal ring infiltrate and other similar conditions such as peripheral ulcerative keratitis.

## Nomenclature

The nomenclature of “corneal ring infiltrates” is ambiguous. In the literature several descriptions might be found: “sterile corneal ring infiltrate”, “infectious ring infiltrate”, “non-infectious ring infiltrate”, “sterile ring”, “immunological ring infiltrate”, “Wessely ring”, “ring abscess”. To clarify “infectious ring infiltrate” refers to a ring of microbial origin, where bacterial/viruses particles are found in the scraped material of the ring. It also correlates with clinical symptoms of infection (hyperemia, pus, anterior chamber activation, decreased vision). “Ring abscess” depicts an infectious ring, particularly suppurative ones. “Immunologic ring” is equivalent to “sterile” and “non-infectious” ring. “Wessely ring” is a type of sterile infiltrate based on type 3 hypersensitivity response marked by antibodies [5].

Of note, peripheral ulcerative keratitis (PUK) is often linked to ring infiltrates with similar pathophysiology of immunological complexes activating complement, but collagenase (metalloproteinases) activation leading to corneal stroma thinning is required in this condition [6].

By definition, the ring affects 360 degrees of the peripheral cornea leaving a clear margin from the limbus. In general, the ring appears more often in the peripheral cornea, where the number of antigen-presenting cells surpasses the central cornea [7]. However, at the disease onset single peripheral infiltrates might be present, then coalescing in a ring-like one [8]. Although corneal ring infiltrates originate from corneal stroma activation, the endothelium might also be affected. Endothelial rings occur more often in fungal than bacterial keratitis [9]. Concomitant deprived epithelium also enforces infectious origin.

## Pathophysiology

Cornea is a transparent structure, deprived from blood and lymphatic vessels. The ring forms typically close to the limbus, close to the conjunctival vessels, where the ratio of antigen-presenting cells (APC) exceeds the density of APC in the corneal center similarly to C1 concentration [7]. Bacterial toxins damage host tissues by activating alternate complement pathway via properdin and C3 stimulation. Chemotactic agents attract polymorphonuclear leukocytes into the cornea [10]. No immunoglobulins (of any class) are found in histopathological samples of infectious rings [11]. Infectious rings form within 24–72 h on average. Immunological rings origin from two ways of complement activation: a) antibodies reacting with corneal particles from damaged tissue- type 3 hypersensitivity (Wessely ring) or b) antibody-independent complement activation. Of note, infectious inflammation might also lead to purely immunological corneal ring infiltrate in late mechanism of hypersensitivity (via release of microbial endotoxins triggering properdin and finally complement activation on antibody-independent way) [12]. Furthermore, exposure of compromised corneal epithelium to saprophytic flora (antigen) might contribute to sterile ring infiltrate formation in contact-lens overwear, corneal foreign body, corneal burns and intoxication, recurrent corneal erosion syndrome, topical anesthetic abuse, post-surface refractive surgery, and in corneal cross-linking.

## Etiology

Tables 1 and 2 depict the potential origins of infectious and non-infectious ring infiltrates. It is important to distinguish the origin from infectious and non-infectious etiologies. Additionally, we need to reiterate that microbial causes may also trigger an immunological ring. Still, the mainstay of primary differentiation is based on clinical signs and symptoms. Patients presenting with decreased vision, pain, pus in conjunctival sac, anterior chamber reaction, epithelial defect, corneal melting and less common- necrosis raise a high suspicion of infectious ulcer [4, 13]. They occur 24–48 h after inoculation and are visible clinically after 2–3 days [14]. Untreated infectious rings lead to centrifugal expansion deteriorating corneal stability. *Pseudomonas aeruginosa* has been proven to impair collagen structure leading to descemetocele or melt instantly (within 7 days) [15]. Sterile rings typically manifest with eye irritation, mild photophobia and decreased vision when corneal structure is damaged (thinning and finally scaring process present) [16]. Immunologic rings slowly expand centripetally and eventually fade. Both etiologies may leave significant corneal thinning and neovascularization. Wessely’s rings form within 10–14 days from the trigger, but may appear within 1–5 days with previous exposure to the antigen [17]. *Acanthamoeba* and *Microsporidium* are the exceptions where immunological ring forms as a result of inappropriate treatment, with > 16 days in Acanthamoeba and even 2 months in Microsporidial keratitis [18, 19].

**Table 1:** Infectious etiology of corneal ring infiltrate (CRI)

Group of disorders	Species	Key differences
Bacteria- Gram-positive	Staphylococcus Streptococcus Bacillus cereus Listeria monocytogenes Nocardia	- 4% prevalence - early presence (24-48 h from inoculation) - associated hypopyon, hyperemia, epithelial defect, decreased vision) - mainly Gram-negative and mixed infections
Bacteria- Gram-negative	Pseudomonas Moraxella Serratia Neisseria Klebsiella Escherichia Proteus Mycobacterium Capnocytophaga Microsporidium	
Fungi	Aspergillus fumigatus Aspergillus flavus Aspergillus niger Fusarium Acremonium	- 1–25% prevalence - yellow or creamy-white - coexistence of patchy, stromal infiltrate - long-lasting (presents till day 7, persists to at least 1 month)
Viruses	Herpes simplex Varicella zoster	
Acanthamoeba	Various Acanthamoeba subspecies	- 30% of cases - presents late (on average 7–14 days from infections’ origin) - greyish - 9–11 times more often than in fungal origin
others	Infectious crystalline keratopathy (Streptococcus mitus)

**Table 2:** Non-infectious etiology of corneal ring infiltrate

Group of disorders	examples
General diseases	Rheumatoid arthritis Cryoglobulinemia Multiple myeloma and other hipergammaglobulinemias Amyloidosis
Ocular diseases	Posterior polymorphous dystrophy
Medications- topical	NSAIDS, topical anaestetics’ abuse
Medications- general	Perifosine
Post-laser	Collagen cross-linking Post-refractive surgery (excimer photoablation)
Foreign body	Corneal foreign body Corneal intacts Corneal burns Insects hair/sting
Toxins	Coral toxins Insects’ toxins Endotoxins (secondary to endophthalmitis)
Trauma	Contact-lens wear Recurrent corneal erosions
Others	Behcet’s disease

## Epidemiology

There is no data on exact prevalence and incidence of the ring infiltrate. However, when the general prevalence of diseases associated with CRI (especially keratitis) is considered some statistics might be indirectly calculated. Mascarenhas reported that 30% of Acanthamoeba keratitis 5% of fungal and 4% bacterial infiltrates present with corneal ring infiltrate at some stage [4]. Bharathi proved the presence of CRI in 1.4% of fungal infections [20]. Several reports of non-infectious ring infiltrates in CL-wearers show 0.5–6% annual risk of symptomatic sterile infiltrate [21, 22]. Post cross-linking CRI were diagnosed in 2–8% of cases [23–25].

Considering that 5% of global population wears contact lenses, they prevail among sterile CRI origins [26]. A study on the prevalence of keratitis showed 0.15% of population affected, with 44% of viral (0.065%), 46% of bacterial origin (0.068%), 10% of fungal (0.015%) in a study by Cao resulting bacterial as a second most common etiology of CRI [27].

Wearing contact lenses increases the incidence of infectious keratitis, sterile corneal ulcer (including ring infiltrate, also called “CLACI”- contact lens associated corneal infiltrates), contact lens peripheral ulcer (CLPU) and CL-induced acute red eye (ang. contact lens acute red eye- CLARE) [28–31]. CLPU and CLARE are mainly associated with extended CL wear. CLPU is manifested with peripheral epithelial full-thickness, regular is shape lesions with co-exisiting corneal stromal infiltrate [28]. CLARE is defined by sudden onset of painful red eye, typically early in the morning, where slit-lamp findings comprise: conjunctival and limbal redness and corneal infiltrate [32].

All abovementioned might be manifested with ring infiltrate. Differentiation relies on clinical criteria. Infectious infiltrates resemble previously described non-sterile infiltrates. Gram (-) bacteria are found more often in CL-related ulcers, with Pseudomonas aeruginosa being the most common origin, with poor final visual outcome [31]. Of note, atypical cases of sterile, CL-linked, infiltrates are described [3]. Tabatabaei reported bilateral deep infiltrate affecting corneal epithelium (epithelial defect and oedema), anterior and deep stroma (dense ring infiltrate) coexisting with pain, redness and discharge. However, difference in epidemiology was described, with higher total incidence in CL-wearers, particularly of Acanthamoeba and fungal origin compared to non-CL-wearers [32]. Of note, factors increasing prevalence of infectious keratitis in CL-wearers are: extended wear, swimming in CL, handwashing, overnight use, low hygiene level, short term of applying CL (< 6 months), male gender, onset in winter, young age, poor hygiene, internet purchase and smoking [31, 32].

Sterile infiltrates in contact lens wearers are noticed more often in hydrogel than silicone lenses. Whereas extended wear increases the risk with odds ratio of 4–5 compared to daily wear, numerous risk factors are coined for all types of lenses: omitted/infrequent disinfection, bacterial contamination of the storage case, initial period of adaptation to CL, prior inflammation related to lenses wearing, smoking, age (< 25 years ld and > 50 years old), limbal redness, corneal staining, high ametropia [31]. Additionally, toxic corneal staining poses a threat of sterile infiltrate also in daily disposable wearers with continuity of risk correlated to area of epithelium defects.

Sterile ring infiltrates in CL wearers is typically located in the superficial part of anterior stroma and resolves within 1–3 weeks. In the majority of cases no final visual acuity deprivation is noted [29].

In summary, the most common infectious origin of CRI is bacterial keratitis and non-infectious cause is contact lens related.

## Infectious causes of corneal ring infiltrate

### Acanthamoeba keratitis

A ring infiltrate is a well-described clinical sign of AK visible in about one third of AK cases [4]. It is located in the corneal stroma where amoebic cysts, polymorphonuclear leukocytes, and activated keratocytes are found. The presence of CRI increases the probability of Acanthamoeba by 11 times compared to bacterial infection and about 9 times compared to fungal etiology. Raghavan added that in mixed infections (Acanthamoeba plus fungi) the risk of ring infiltrate presence is even higher and its morphology alters (into yellowish with hyphate edges) [33]. Clinically CRI in AK is classically greyish [33]. Typically CRI appears after few days (7–14 days, 100% by the day 7 in the Raghavan study) from the inoculation and is persistent for more than a month. Marcarenhas hypothesizes that the presence of corneal ring infiltrate reflects long-lasting inflammation as the Acanthamoeba is properly diagnosed relatively late compared to other infectious etiologies [4]. Notably, Dahglren et al. writes that if a ring infiltrate is present the clinicians set the diagnosis of AK more often [34]. Carnt et al. analyzed 194 cases of AK in the United Kingdom looking for predictors of poorer outcome. She concluded that scleritis and ring infiltrate were the most prominent and persist when the treatment was suboptimal [35]. The presence of Wessely ring in AK has also been reported [36]. Holland depicted six cases of peripheral CRI occurring several months after primary infection. They were localized in the anterior stroma or subepithelium resembling viral/chlamydial infection and resolving with topical corticosteroids treatment [36]. Although the exact pathophysiology is unknown, the authors speculate that the ring formation might be linked to residual Acanthamoeba antigens released from the cornea or hypersensitivity reaction to long-lasting treatment (propamidine isethionate) [7].

### Bacterial infection

A corneal ring infiltrate may be also found in bacterial infections. Typically, CRI in these cases is associated with hyperemia, hypopyon, decreased vision as a part of corneal ulcer appearance. They are present after 24–48 h of inoculation. Microbial toxins, derivatives of damaged cells and complement attract inflammatory cells. However, immunological reaction on bacterial epitopes is also a well-described mechanism for the presence of CRI in infectious keratitis. The pathophysiology of the latter is based on late response to the pathogen and typically occurs relatively late in infectious keratitis (on average 10–14 days of primary infection).

The most common microbials associated with CRI are: *Staphycoccus aureus* and *Pseudomonas aeruginosa* and *Serratia marcescens*. The list of Gram negatives also include: *Escherichia, Klebsiella, Proetus, Neisseria, Moraxella, Microsporidium, Capnocytophaga and Mycobacterium* [10, 37]. Of note, CRI is often present with multi-drug resistant bacterial keratitis.

Gram-negative bacteria are present with CRI more often than Gram-positive bacteria. A ring develops earlier (typically after 24–72 h) in bacterial infection, especially Gram-negative, than in viral, fungal and Acanthamoebal origin. On average Gram-negative ring infiltrates are deeper and denser than those of Gram-positive etiology. Gram-negative rings consist of more multiple polymorphonuclear leukocytes, densely packed at the periphery of the ring. Furthermore, Gram-negative bacteria release abundant amount of proteases leading to collagen fibers degradation and stromal necrosis contributing to ring infiltrate formation [5].

*Staphylococcus aureus* is one of the most common Gram-positive bacteria associated with corneal ring infiltrates. Its prevalence is relatively high due to triggering CRI in both mechanisms- as a typical infectious ring, and secondary immunological reaction. That may be explained by the presence of Staphylococcus saprophytic flora of the eyelids margins and conjunctival sac. Furthermore, Staphylococcus aureus-related ring infiltrate is also associated with marginal keratitis, secondary to chronic blepharoconjunctivitis, where the bacteria act as an initiator of an antigen–antibody reaction cascade, then complement activation and neutrophils infiltration. Often, CRI in Staphylococcus aureus predisposes to resistance to medical therapy. Marginal keratitis per se may also coalesce in greyish CRI located circumferentially 1 mm from the limbus and resolve with steroid topical treatment [13].

*Serratia marcescens* is a facultative, aerobic, Gram-negative bacillus from Enterobacteriacae genre. Ocular involvement might be manifested as keratitis, conjunctivitis, scleritis or endophthalmitis. Chaidaronn presented a HIV positive patient diagnosed with keratitis with hand motions vision, hypopyon, conjunctival discharge, deep and broad 360 degrees peripheral corneal ring infiltrate as a Serratia infection [38].

Atypical bacteria like Moraxella rarely invade cornea but should be especially suspected in malnourished, alcoholic patients suffering from multiple general diseases. Moraxella presentation ranges from mild blepharoconjunctivitis to severe keratitis [5]. Barash was the first to describe Moraxella keratitis with central ring (day 3) fading with fortified antibiotics treatment and presenting with another ring (peripheral to the previous one, presumably autoimmunological one) [5].

Another bacteria- Streptococcus mitis is a causative factor of separate disorder: infectious crystalline keratopathy. It might be asymptomatic or giving typical bacterial keratitis symptoms. Slit-lamp examination typically reveals whitish/grayish branching fern-like crystalline opacities in anterior stroma, often coalescing to ring-like structure [39].

#### Bacillus cereus

Bacillus cereus is a Gram positive, aerobic bacteria typically affecting post-traumatic eyes. The bacteria cause ring shape infiltrates with coexisting hypopyon. This constellation is suggestive to endophthalmitis, typically post-traumatic endophthalmitis. A fulminant course of the disease leads to very low vision, evisceration or enucleation in more than 75% of the patients [40, 41].

#### Nocardia

Nocardia is a Gram positive aerobic bacteria, that forms branching filaments resembling fungal hyphae. It grows slowly on the medium, typically within 2–5 days, but 2–3 weeks incubation is needed to rule out late growth. The natural reservoirs of Nocardia are soil and oral mucosa. Most of the ocular infections are acquired through soil contamination (after trauma) or inhalation. Low virulence contributes to infecting mainly immunocompromised patients (elderly, children, HIV + patients). The Nocardia genus includes 85 species and the majority of them requires combined antibiotic therapy. Beaded wreath appearance or confluent ring infiltrate are described [42].

#### Infectious endophthalmitis

Corneal ring infiltrates as a part of endophthalmitis picture were described with numerous infective agents (Pseudomonas aeruginosa, Staphylococcus aureus), but the most severe cases are associated with Bacillus cereus. Basak et al. published a case report of panendophthalmitis after phaco-DSAEK procedure originating from Bacillus cereus infection that finally led to evisceration due to its fulminant course [43].

Endogeneous endophthalmitis is defined as an intraocular infection secondary to other part of the body infection spread haematogenously. In 2017 Akkach et al. published a first case of endogenous endophthalmitis being manifested with a dense, 3 mm wide, corneal ring infiltrate [44]. This particular patient presented with a severely decreased vision (light perception), large hypopyon and 210 degrees corneal ring infiltrate.

### Fungal infections

Fungal keratitis carries one of the worst prognoses for final visual outcome among corneal ulcers. A need for surgical intervention (mainly therapeutic corneal transplant) reaches 25–40% [45, 46]. Moreover, fungal corneal ulcers pose significant threat of enucleation [47]. They are often misdiagnosed as a HSV (significantly when keratoneuritis is present) or Acanthamoeba keratitis (especially when ring infiltrate is present). Thus, a meticulous history and examination should be performed to avoid misdiagnosis. The typical signs of fungal infection in slit-lamp examination are serrated borders, raised slough, satellites/multifocal lesions, dry texture of slough, coexistent hypopyon and color (other than yellow) [9, 48]. Multimicrobial ulcers worsens the diagnosis and occur in around 4–10% of fungal keratitis (Acanthamoeba or bacterial coexistence) [33, 49]. According to the available literature, a corneal ring infiltrate is present in 1–25% of fungal keratitis [20]. A corneal ring infiltrate in fungal and mixed keratitis, found in around 5% of cases, has a slightly different appearance: more often yellowish or creamy white with associated central patchy stromal infiltration [33]. In Acanthamoeba-fungal coinfection additional features are found: hyphate edges and dot-like infiltrates emanating from the ring infiltrate. Interestingly, when the infection prolongs, the ring is often replaced a uniform circumscribed infiltration, no-longer resembling classical ring infiltrate. However, study of Raghavan proved that the ring is present by the day 7 of the onset and in 100% of cases persist till at least one month [33].

There is no data available supporting different appearance of CRI in different fungus genres. Typically, a ring forms 1 mm from the limbus leaving a clear zone of transparent cornea, has white/yellow colour and 2 mm of width and variable depth [33, 50]. Aspergillus fumigatus might be an exception and resemble Nocardia with wreath-pattern ring infiltrate [51].

However, considering higher prevalence of fungal than Acanthamoeba keratitis, medical doctors should always remember first about potential fungal keratitis as an etiologic factor of CRI. In 2021 Ahmadikia published robust meta-analysis of corneal ulcers noticing that around 23% of cases are of fungal origin [49].

A deep-learning algorithm to differentiate the origin of keratitis, CRI included, was also implemented but a statistical difference was found between artificial intelligence and an experienced ophthalmologist. The study involved a robust number (669) of slit-lamp photographs of bacterial and fungal corneal ulcers [52]. Thus, many authors underline the value of repeating scrapes when persistent non-healing corneal ulcer is present with no growth on the first corneal sample analysis.

### Viral infections

Both infection and postinfectious immunological reactions play a role in pathophysiology of corneal ring infiltrate formation in viral infections. The majority of viruses present with late ring in immunological reaction to the antigen. Theoretically, all viruses infecting the anterior surface may cause CRI, eg. Herpes simplex virus, Varicella zoster, cytomegalovirus, Epstein-Barr virus, adenoviruses. However, herpes viruses (Herpes simplex and Varicella zoster) predominant. Apart from primary infection we should remember that 40% of herpetic keratitis recur in 2–5 times in lifespan [53].

#### Morphology of corneal ring infiltrate in viral infections

Mondino reported a dense pannus with incomplete ring infiltrate with 4 herpes zoster ophthalmicus patients without disciform keratitis, interstitial keratitis nor scleritis [54]. The ring was located in anterior and mid-stroma. Khan described the case of late CRI after chickenpox infection in 7-yo girl presenting as mid-peripheral stromal dense deep ring infiltrate with mild stromal thinning (< 25% of total dept of the ring) causing irregular astigmatism (7.63D). It was diagnosed 2 months after primary chickenpox infection with palpebral lesion [55]. Six-week course of topical prednisolone and oral valacyclovir was sufficient to clear the cornea, but significant thinning persists.

Pandit et al. reported a case of atypical- triple ring infiltrate in a patient with presumed HSV keratitis. Swabs, culture and IVCM were negative for either: bacteria, HSV, or fungi infection. The patient had been experiencing pain and photophobia for 3 days prior to referral. Interestingly, the patient had been suffering from cold sores on the skin of his mouth for his whole adulthood and on his eyelids 7 years ago. Empirical treatment with oral acyclovir and topical 1% prednisolone acetate improved vision to 20/20 at day 25. Complete 360 degrees 2.5 mm in diameter ring infiltrate was located in inferonasal quadrant and co-existence of peripheral 180 degrees and the third- 90 degrees close to the limbus.

Altan-Yaycioglu reported bilateral central corneal ring infiltrate on the borders of disciform keratitis of presumably adenoviral etiology. Rings in that case are associated with widespread subepithelial corneal infiltrates. They resolve upon topical treatment with prednisolone acetate [56].

## Non-infectious causes of corneal ring infiltrate

### Drugs

#### Topical drugs

Topical 0.5% proparacaine is an anaesthetic widely applied in ophthalmology. However, its chronic use is contraindicated due to potential toxicity. Mean time to impair toxic corneal effect ranges from 1 to 30 days of use [57]. Clinical characteristic of toxicity comprises epithelial defect, persistent included, stromal infiltrate, keratic precipitates, endothelial cell loss, corneal oedema, corneal stromal melting and finally perforation [58]. Prolonged proparacaine administration impairs tear production, mucous stability and corneal nerves function, leading to excess tear evaporation. Exposed epithelium cells are shed acting as epithopes for inflammatory response, simultanously allowing stromal keratocytes to activate leading to stromal infiltrate, edema, and melting [59].

Although side effects of both, topical and generally administered NSAIDs are well described, little is known about them being the risk of corneal ring infiltrate. Topical NSAIDS (indomethacin, ketorolac and diclofenac) might cause prolonged mydriasis, impaired epithelium healing, corneal erosions, contact dermatitis, decreased corneal sensitivity leading to superficial punctual keratitis, stromal keratitis, lacrimal canalicular obstruction and corneal melting [60–62]. Of note, systemic diclofenac was also reported to cause peripheral corneal infiltrates [63]. A large body of evidence implies that corneal side effects of NSAIDS occur with additional triggering factor, e.g. cataract or refractive surgery [61, 64]. Moreover, the majority of them occur in patients with previous dry eye syndrome. The pathophysiology is similar to the topical anesthetics described above. The primary goal in terms of treatment it to stop the NSAIDs drops and halt the melting process [61, 65].

#### General drugs

Perifosine is an anticancer medication, PI3K/Akt/mTOR inhibitor, registered in macroglobulinemia Waldenstromii, GISTs and metastatic brain tumor [66]. Keenan described a patient with corneal ring infiltrate appearing 5 months of 150 mg dose of perofosine daily intake [67]. The clinical presentation was relatively severe with mixed hyperemia, rapidly progressing deep stromal ring infiltrate and overlying epithelial defect. Of note, decreased corneal sensation was present from the symptoms’ onset. The infiltrates finally progressed to limbal and scleral thinning retained by aggressive oral antiinflammataory therapy (mixed glicocortycosteroids and cyclophosphamide). Finally, the patient was severely visually impaired due to total corneal opacification and neovascularization.

Another five cases of CRI due to perifosine therapy were described in GIST patients on mixed imatinib-perifosine therapy with 100 mg daily or 900 mg weekly dose [68]. They resemble peripheral ulcerative keratitis and last for 1–3 months before appropriate diagnosis was set.

Two patients with bilateral and three with unilateral CRIs presents potentially infectious symptoms (decreased visual acuity, redness, photophobia, irritation) with no improvement on antibiotics, with aggreviation of symptoms and progressive corneal thinning in majority of cases. Perifosine discontinuation or topical or mixed (topical with oral) corticosteroid therapy alleviate pain and other symptoms allowing the cornea to heal.

### Collagen cross-linking

Several reports on both infectious and, more often, sterile ring infiltrates following collagen cross-linking (CXL) are available in the literature [69–72]. The sterile infiltrates occur in 0.9–7.6% of CXL patients [25]. They differ in size, location and depth but typically appear in the anterior stroma of peripheral cornea, zone of removed epithelium (irradiation zone of 9 mm) [73].

The non-infectious rings occurring after cross-linking procedures thrived discussion about its potential pathophysiology. Hypersensitivity to palpebral flora, mainly Staphylococcus, NSAIDS toxicity, hypersensitivity to riboflavin or UVA light, epithelial damage (triggering migration of inflammatory cytokines and cells), keratocytes’ cytotoxicity are all possible reasons [23, 74, 75].

The latter (keratocytes hypersensitivity leading to CRI formation) raises special interest as it is well known that keratocytes apoptose to 300 um depths after CXL causing rearrangement of tissue and oedema. Apoptosis of keratocytes modifies antigens on its surface being a potential target to the immune response [71]. Placing a contact lens directly after the procedure may also trigger hypoxia and subsequent inflammation response. Direct toxicity of UVA light and riboflavin cause early infiltrates (1–7 days) while apoptotic mechanism is rather responsible for late changes (up to 3 months) and disappear after topical steroid treatment [76].

### General diseases

Many autoimmunological diseases may manifest with a sterile ring infiltrate such as paraproteinemias (multiple myeloma, Waldenstrom macroglobulinemia) and rheumatoid arthritis. Case reports on lymphoma, Sjogren disease and systemic lupus erythematosus also exist.

#### Rheumatoid arthritis

Autoimmunological connective tissue diseases present various ocular problems: dry eye syndrome, sterile keratitis, scleritis, peripheral ulcerative keratitis (PUK). All of the above, might occur when no underlying disease activity is present. Sterile corneal infiltrate, ring infiltrate included, are less frequent than PUK and not associated with uveitis, scleritis nor conjunctivitis contrary to PUK [77]. Pathophysiologically, overexpression of proinflammatory cytokines (IL-6, TNF-alfa), reactivity to the antigens of MHC class II, monocytes and histiocytes, ultrastructural collagen alterations and biochemical changes leading to corneal melt were observed. Increased number of T-helpers lymphocytes leads to B-lymphocytes activation and abnormal production of antibodies [77]. They form immune complexes and store in peripheral vessels around the limbus, crossing 0.5 mm of the cornea in vascular anastomosis, what activates the complement pathway leading to acquisition of the neutrophils, monocytes, histiocytes (and even eosinophils). The cells release enzymes destructing corneal stroma (mainly collagenases) [78]. Imbalance of metalloproteinases MMP2 and MMP-9 levels on the eye surface may further worsen the corneal status leading to its thinning. Interestingly all ocular changes response well to cyclosporine [79]. However, they progress rapidly leading often to corneal transplant [77].

#### Multiple myeloma and other paraproteinemias

Multiple myeloma is a malignant monoclonal protein proliferation affecting numerous organs, including the eye. The most common signs affect the cornea and might be divided into crystalline keratopathy, immunotactoid keratopathy and vortex keratopathy. However, corneal haze, persistent corneal oedema, repeated subconjunctival hemorrhages, peripheral ulcerative keratitis, prominent corneal nerves and band keratopathy are also noted [80, 81].

Corneal involvement is calculated on 1 in 100 patients with paraproteinemias [82]. Immunoprotein corneal deposits typically occupy anterior stroma, but epithelium, posterior stroma, endothelium, Bowman and Descemet’s membrane involvement has also been reported [83–86]. Ring infiltrate morphology might be linked to “immunotactoid deposition”- typical tissue structure found in paraproteinemias- primarily described in renal biopsies and the name transferred to other organs. By definition it consists of organized microtubules deposits of IgGκ [86, 87]. Immunofluorescence shows an accumulation of IgG, C3 and kappa light chains. Furthermore, also crystalline deposits, comprising pleomorphic, osmophilic, intracellular crystals, might be found in anterior stroma and perilimbal conjunctiva [87]. Of note, the CRI might present with petaloid configuration in retroilumination and coexist with thinned epithelium (to 25–30 um) [84].

Laboratory work-up comprises of serum protein electrophoresis, C-reactive protein, protein level, urea and creatinine level, glucose, electrolytes, liver function, lipid profile, calcium profile [83]. Paladini proved that in IVCM might be considered the gold standard ancillary test to suspect and monitor hipergammaglobulinemias in cornea [88]. However, only pathomorphological examination provide unquestionable diagnosis.

Empirical treatment starts with topical steroids showing promising effect, but the opacities return after cessation. General targeted chemotherapy reduces corneal haze but still penetrating keratoplasty is of need in selected patients. Recent reports suggest that gas permeable contact lenses might be viable alternative for symptomatic treatment of irregular astigmatism induced by corneal changes.

#### Cryoglobulinemia

This immunological disease is mainly associated with viral infection, mainly hepatitis C virus, but might be also found in otherwise healthy people. Cryoglobulins are immune complexes consisting of anti-viral immunoglobulins and IgMs being a product of extensive B-cells activation triggered by a virus. Cryoglobulins may cause a pure inflammatory response or medium vessel vasculitis, both giving occlusive disease. Ophthalmologists should look mainly at anterior segment abnormalities as cryoglobulins precipitate in low temperatures (cornea has about 2 deg lower temperature than the rest of the body) [89]. Thus, keratitis, commonly ulcerative keratitis, and necrotizing anterior scleritis are the most common ocular signs of the disease. General treatment with either immunosuppresive or immunomodulatory drugs is needed to halt the disease (oral glucocorticosteroids, cyclophosphamide, methotrexate or cyclosporine).

#### Behcet’s disease

Behcet’s disease is an inflammatory disorder manifesting mainly with mucocutaneous ulcers, uveitis and arthritis [90]. Interestingly, bilateral corneal ring infiltrates following chronic conjunctivitis was reported in a patient with 6-years history of Behcet’s disease [91]. The authors hypothesized that persistent conjunctival inflammation may lead to limbal vasculature activation and release of immunological complexes activating complement via properdine.

## Ancillary testing in corneal ring infiltrate

### Corneal material sample testing

There are two main approaches to solve the problem of ring infiltrate’s origin. One of them requires taking corneal scrapes as a golden standard and treating as a sterile only when not proven otherwise [92]. Another one, suggested by McLeod, allows us to choose between three options considering cost-effectiveness: 1) following the general rule and perform scrapes and its analysis to every ulcer, 2) scrape only suspects of severe infiltrates (hypopyon, affecting central axis, thinning of at least 50% of the normal thickness) with high suspicion of non-bacterial etiology, 3) scrape and introduce empirical treatment, perform material examination only when empirical treatment is ineffective [93]. On the other hand, patients with well-known risk factors for sterile infiltrates and non-compromised epithelium might be treated directly as a non-infectious ulcer. In the literature there are scales aiming to stratify the probability of infectious versus sterile infiltrate probability. They rely mainly on clinical features of the ulceration [94]. However, all of those systems lack evidence-based medicine evaluation.

Corneal biopsy remains also an option for ambiguous cases, especially with inconclusive scrapes results and further deterioration despite best-tailored treatment [95]. Another ancillary test- PCR (polymerase chain reaction)- targets selected microorganisms (mainly bacteria, viruses, Acanthamoeba and fungi) and represents similar sensitivity and specificity to corneal scraping. However, it requires targeted primers for each suspected etiology leading to high costs [96]. Finally, next generation sequencing (NGS) may play complementary role, especially in treatment-resistant infiltrates [97].

### Anterior segment optical coherence tomography (AS-OCT)

Anterior segment optical coherence tomography might quantify the corneal ring infiltrate providing an information on the depth, width and margins of the infiltrate, as well as to monitor the corneal thinning [98]. It can also assess posterior stroma/endothelium/anterior chamber especially when the view is limited in corneal edema [98]. AS-OCT may guide the distinction between corneal edema, active infiltrate and scar leading to a more accurate treatment. Despite being a useful tool in measuring the infiltrate, it does not allow to differentiate between the origins. However, clinicians adopt AS-OCT to assess healing process and monitor the corneal thickness. Jansen reported a mean of 10 days for the infiltrate to resolve, defined as transition with diffused margins to distinct ones [99]. Moreover, defined margins are believed to be precursor of clear resolution or scarring.

### In Vivo Confocal Microscopy (IVCM)

IVCM is a non-invasive, in-vivo ancillary test with resolution of 5–7 um. Thus, IVCM detects only the pathogens exceeding this size- mainly Acanthamoeba and fungi. Highest sensitivity and specificity of IVCM concerns Acanthamoeba with following signs: double wall cyst, signet ring, trophozoite, bright spots and perineural infiltrates [100, 101]. Aspergillus and Fusarium, representing filamentous fungi, are suspected when hyphae-liked structures are visualised (elongated hyper-reflective lines, branching under 45 or 90 degrees) [102]. CMV-suspected endothelitis with “owl-like” morphology might also be suspected based on IVCM characteristic features [103]. Lastly, specific deposits (“immunotactoid deposits”) visible in IVCM might be linked to some paraproteinemias [87].

There is no EBM-based body of evidence supporting IVCM analysis in other potential origins of ring infiltrate. However, cellular processes of corneal tissue (keratocytes activation, immune cells concentration, reorganization of cellular structures) might be assessed via IVCM [104].

### Laboratory work-up

Laboratory work-up is hardly ever mentioned in differential diagnosis of corneal ring infiltrate. However, it seems to play a role in scrapes negative patients with unknown risk factors for sterile infilatrates. Liu et al. recommends a panel consisting of: erythrocyte sedimentation rate, C-reactive protein, hematology analysis, antinuclear antibody, rheumatoid factor, antineutrophil cytoplasmic antibodies, tuberculosis (TB) assay, TORCH infection [105].

## Treatment

### Infectious ring infiltrate

Microbial keratitis presenting with ring infiltrate should be managed as infectious corneal ulceration. In bacterial keratitis, topical antibiotics either fluroquinolone monotherapy or aminoglycoside-cephalosporin polytherapy are the mainstay of treatment [106, 107] with a frequent of hourly drops for the first 24 h with subsequent tapering of the dose.

There is no EMB on oral antibiotic treatment applied in some centers aiming to inhibit metalloproteinases and stop corneal melting.

The gold standard of antifungal treatment is still 5% natamycin proven by the Mycotic Ulcer Treatment Trial (MUTT I) [108, 109] When natamycin availability is limited, combined therapy of topical amphotericin B and topical voriconazole should be considered.

Viral infiltrates require mainly topical antivirals. Guidelines differ across the world: with topical acyclovir (effective against HSV, VZV) or ganciclovir (effective against HSV, VZV and CMV) in Europe and trifluridine in USA [110, 111]. Topical corticosteroids (prednisolone phosphate) might be added in stromal HSV keratitis [112]. Oral acyclovir (5 × 400 mg in HSV and 5 × 800 mg in VZV) or valacyclovir (3 × 1 g in HSV, VZV and CMV) are either adjuvant treatment in stromal viral changes or replacement treatment if topical drugs’ toxicity is unwanted [113].

Acanthamoeba keratitis treatment involves either a combination of biguanides (mainly 0.02% polyhexamethylene-biguanide – PHMB) and diamidines (mainly 0.1% propamidine isethionate) or 0.02% chlorhexidine [114].

### Sterile ring infiltrate

Topical glucocorticosteroid (CS) therapy is a mainstay of treatment in non-infectious ring infiltrates. There is no global consensus on type, dosage and time of appropriate treatment. The literature shows 1–8 weeks of CS regimen of 1% prednisolone acetate, 0.1% fluorometholone, or 0.5% loteprednol etabonate. If there is a contraindication for steroid treatment, calcineurin inhibitors might be considered. Topical 0.05% or 0.1% cyclosporine and 0.03% tacrolimus have been proven to heal sterile corneal infiltrates and further reduce haze formation [115].

## Conclusions

The corneal ring infiltrate is widely linked to Acanthamoeba infection. However, the literature shows a wide variety of potential causative agents, from bacteria, fungi, viruses through immunological processes (drugs toxicity, CL wear, general diseases) to foreign bodies and trauma. Unfortunately, no pathognomonic symptoms nor signs related to CRI origin have been found so far. The precise differentiation remains challenging even though new technologies are applied (As-OCT, IVCM, artificial intelligence). An existing large body of evidence suggest that corneal ring infiltrate should be scraped and treat empirically with anti-infectious agents until proven otherwise. Laboratory tests are ordered in scrapes negative patients with no co-existing risk factors for sterile infiltrates. Infectious rings, either confirmed in corneal sample result or suspected clinically and improving on proper medication, need to be treated as microbial keratitis. Sterile infiltrates fade within time in general with no treatment, but topical steroids facilitate resolution of Wessely’s ring. Non-infectious etiology recurs more often, resulting in scars and melting.

Further research is of need to guide the clinicians with most probable etiology and proper management of CRI.

## Data Availability

Not applicable.
